# Synthesis and in vitro activity of novel 2-(benzylthio)-4-chloro-5-(1,3,4-oxadiazol-2-yl)benzenesulfonamide derivatives

**DOI:** 10.1007/s00706-012-0732-6

**Published:** 2012-03-02

**Authors:** Kamil Brożewicz, Jarosław Sławiński

**Affiliations:** Department of Organic Chemistry, Medical University of Gdańsk, Al. Gen. J. Hallera 107, 80-416 Gdańsk, Poland

**Keywords:** Acylsulfonamides, 2-Mercaptobenzenesulfonamides, Antitumor agents, Phase-transfer catalysis, Heterocycles

## Abstract

**Abstract:**

Two series of novel 4-chloro-2-(benzylthio)-5-(1,3,4-oxadiazol-2-yl)benzenesulfonamides and their *N*-aroyl derivatives have been synthesized and evaluated for in vitro anticancer activity against the full NCI-60 cell line panel. Most of the compounds exhibited antiproliferative activity. Among them a compound bearing an *N*-(thien-2-ylcarbonyl) moiety showed broad-spectrum activity with 50% growth inhibition (GI_50_) values in the range of 2.02–7.82 μM over 50 cell lines.

**Graphical abstract:**

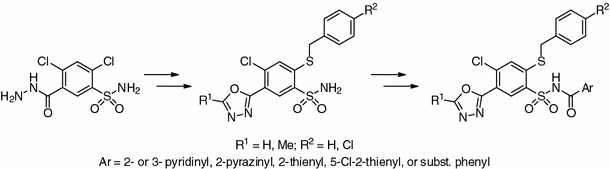
.

## Introduction

**Fig. 1:**
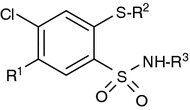
MBSA scaffold [20, 21]

Aryl- and heteroarylsulfonamides are an important class of therapeutic agents in current medicinal science [1]. Various arylsulfonamides have been reported to possess anticancer [2–6] and/or anti-human immunodeficiency virus (HIV) properties [6, 7]. Our systematic studies on the synthesis of 1,4,2-benzodithiazine 1,1-dioxides and their subsequent transformations into 2-mercaptobenzenesulfonamide (MBSA) derivatives (Fig. 1) having a variety of heterocyclic ring systems or acyclic polynitrogen moieties at the sulfonamide functionality resulted in promising anticancer [8–13], HIV antiviral [14–16], or antibacterial agents [17] as well as potent inhibitors of transmembrane cancer-associated carbonic anhydrase isozymes hCAIX and hCAXII [18, 19].

**Fig. 2:**
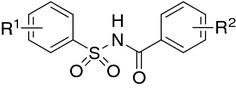
Acyl sulfonamide antiproliferative (ASAP) scaffold [26]

**Fig. 3:**
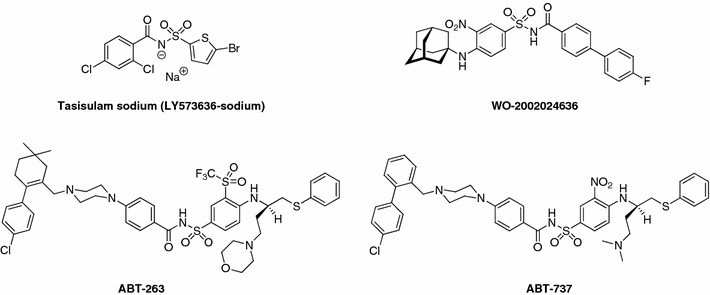
Tasisulam sodium (LY573636-sodium): clinically evaluated (phase II/III in metastatic melanoma) antitumor *N*-acylsulfonamide; pan-Bcl family inhibitors targeting Bcl-2, Bcl-w, and Bcl-x_L_: WO-2002024636, ABT-737, and ABT-263 [23–25]

A number of structurally novel *N*-acylbenzenesulfonamides have recently been reported either as potent antitumor agents against a broad spectrum of human tumor xenografts (colon, lung, breast, ovary, and prostate) in nude mice [22] (Fig. 2) or clinically investigated drug candidates with cytostatic activity against malignant tumors such as Eli Lilly’s tasisulam sodium [23] or Abbott’s WO-2002024636, ABT-737 [24], and ABT-263 [25] (Fig. 3).

This led us to an assumption that expansion of the series of 2-mercapto-*N*-acylbenzenesulfonamide potential anticancer agents, in which groups of varying size and electronic properties are placed at positions 2, 5, and *N*- of the benzenesulfonamide ring, may shed light on the structural features contributing to the biological activities.

## Results and discussion

### Chemistry

Several methods for synthesis of 2-mercaptobenzenesulfonamides are known. The simplest and most efficient method employs the ring-opening reaction of preformed 3-mercapto-1,1-dioxo-1,4,2-benzodithiazine derivatives under alkaline conditions [27]. Alternatively, access to 2-mercaptobenzenesulfonamides is provided by direct reaction of 2-halogenobenzenesulfonamides with sodium polysulfide (Na_2_S_*x*_) [28] or conversion of 2-aminobenzenesulfonamides via diazonium salt decomposition utilizing disodium sulfide (Na_2_S) or potassium ethyl xanthate [28–30]. Herein, we report a direct synthetic route to novel 4-chloro-2-benzylthiobenzenesulfonamides and their *N*-acylated derivatives. Due to our ongoing research in the field of biologically active 2-mercaptobenzenesulfonamides with five-membered rings incorporated in 5-position of the MBSA scaffold [9], we choose 1,3,4-oxadiazole as our model heterocyclic residue.

**Scheme 1:**
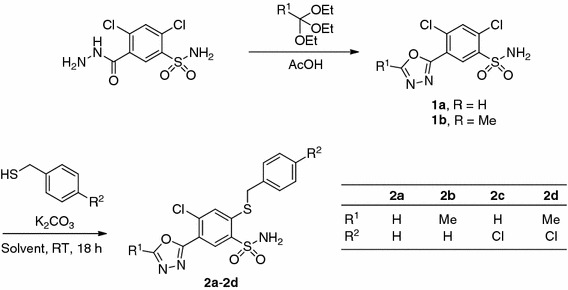

The expected 1,3,4-oxadiazoles **1a**, **1b** were conveniently prepared in good yields by the reaction of 2,4-dichloro-5-sulfamoylbenzhydrazide [31] with orthoesters in refluxing glacial acetic acid (Scheme 1).

We found that 2,4-dichloro-5-(1,3,4-oxadiazol-2-yl)benzenesulfonamide (**1a**) under standard conditions (BnSH/K_2_CO_3_/DMF (*N*,*N*-dimethylformamide)/RT) undergoes a selective S_N_Ar addition–elimination reaction in 2-position. Moderate yields (14–58%, Table 1, entries 1–4, 6, and 8) of this reaction led us to optimize the conditions. Higher yields were observed when tetrabutylammonium bromide (TBAB) was used as a phase-transfer catalyst, especially in acetonitrile/water (300:1, v/v) reaction environment (Table 1, entry 9). Slight decrease of substrate conversion was observed in the absence of argon atmosphere (Scheme 1).

**Table 1:** Reaction of 2,4-dichloro-5-(1,3,4-oxadiazol-2-yl)benzenesulfonamide (**1a**) with benzyl mercaptan and optimization of the reaction conditions

Entry	Solvent	BnSH/mmol	K_2_CO_3_/mmol	Yield^a^/%
1	EtOH	1.0	1.2	Trace
2	DMF	1.0	1.2	32
3	DMF	2.0	2.2	27
4	DMF	1.0	2.2	41
5	DMF/H_2_O	1.0	2.2 (cat.)^b^	55
6	DMSO	1.0	2.2	14
7	DMSO/H_2_O	1.0	2.2 (cat.)^b^	33
8	MeCN	1.0	2.2	58
9	MeCN/H_2_O	1.0	2.2 (cat.)^a^	81

Reaction conditions: 5 cm^3^ solvent at room temperature (ca. 25 °C) under argon atmosphere

*DMSO* dimethylsulfoxide

^a^Isolated yield of **2a**

^b^(*n*-Bu_4_ N)^+^ Br− (0.01 mmol)

**Scheme 2:**
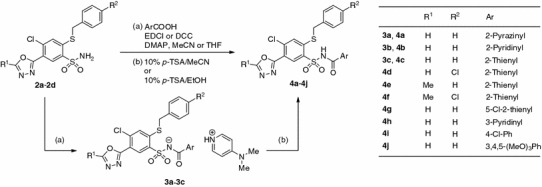

The desired *N*-acylsulfonamides **4a**–**4j** (Scheme 2) were prepared by carbodiimide-mediated coupling of aromatic carboxylic acids with sulfonamides [32–34] promoted by 4-(*N*,*N*-dimethylamino)pyridine (DMAP) in the appropriate solvent. In some cases crystalline 4-(*N*,*N*-dimethylamino)pyridinium *N*-heteroaroylsulfonamidates (**3a**–**3c**) were isolated and characterized, which by treatment with 10% (w/v) ethanolic *p*-toluenesulfonic acid (*p*-TSA) solution were converted to the desired *N*-acylsulfonamides **4a**–**4c**.

### In vitro biological activity

**Fig. 4:**
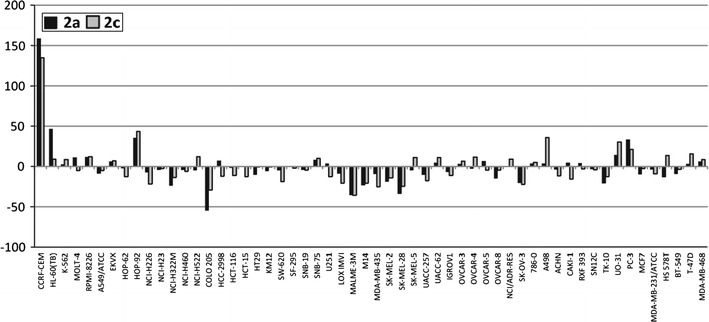
Differential cytotoxicity graph for **2a** and **2c** revealing NCI-60 panel selectivity/resistance pattern expressed in % growth. Sulfonamides **2a** and **2c** show significant selectivity toward CCRF-CEM human T cell lymphoblast-like cell line. For each agent the difference between mean % growth and % growth of each cell line for that agent is determined, to yield positive values for cell lines more sensitive than average (*bars* projecting above the *horizontal* axis) and negative values for cell lines less sensitive than average (*bars* projecting below the *horizontal* axis). Mean graph midpoint (the origin of the abscissa) for **2a** is 98.22% and for **2c** is 92.12%

Compounds **2a**–**2d** and **4a**–**4j** submitted to National Cancer Institute (NCI) were evaluated for their in vitro anticancer activity. Sulfonamides **2a** and **2c** showed significant selectivity toward leukemia cell line CCRF-CEM (Fig. 4), whereas **2d** appears to be substantially inactive.

HOP-92, non-small cell lung cancer, and renal cancer A498 cell lines reveal some insight into structure–activity relationship (SAR). Cytostatic activity of **2a**–**2c** toward those cell lines increases when CLog*P* and calculated molar refractivity (CMR) of the compound increase (Table 2).

**Table 2:** CLog*P* and CMR molecular descriptors of **2a**–**2d**

Compd.	Growth (%)		CLog*P* ^a^	CMR^a^
	HOP-92	A498		
**2a**	62.58	94.43	1.86852	9.5054
**2b**	57.35	59.49	2.13752	9.9692
**2c**	48.61	56.18	2.58152	9.9968
**2d**	84.54	91.46	2.85052	10.4606

SAR based on HOP-92 and A498 cell line screen at 10 μM concentration of the test agent

^a^Molecular descriptors calculated using BioByte software package [35]

Over a series of *N*-(thien-2-ylcarbonyl)benzenesulfonamide derivatives (**4c**–**4g**), substitution on the heterocyclic (**4e**, **4f**: R^1^ = Me) or benzylthio (**4d**, **4f**: R^2^ = Cl) moiety decreases activity significantly. It seems interesting that closely related six-membered *N*-heteroaroyl derivatives (**4a**, **4b**, and **4h**) showed no activity, which renders **4c** as a lead for further optimization.

Compound **4c** (NSC 754633) which satisfied predetermined threshold inhibition criteria was selected for the NCI five-dose (0.01–100 μM) assay and exhibited remarkable anticancer activity against most of the tested cell lines representing nine different subpanels (Table 3). Only NCI/ADR-RES (adriamycin-resistant cell line) expressing high levels of MDR1 and Pgp-170 glycoprotein [36, 37] was found to be insensitive at the highest tested concentration (100 μM). The obtained data revealed some subpanel sensitivity toward renal, central nervous system (CNS), and breast cancer cell lines (subpanel selectivity ratio: 1.04–1.46). The CNS cancer subpanel showed highest sensitivity with mean GI_50_ value of 3.24 μM and mean concentration causing total growth inhibition at 12.68 μM level. It is worth mentioning that the cytotoxic effect of **4c** was less pronounced in the leukemia subpanel [50% lethal concentration (LC_50_) for all tested leukemia cell lines >100 μM]. A relatively large difference in mean cytostatic (mean-graph GI_50_ = 4.27 μM) and cytotoxic (mean-graph LC_50_ = 58.88 μM) indicators could be projected to potential low toxicity against normal cells resulting in a broad therapeutic index.

**Table 3:** In vitro antiproliferative data (μM) for **4c** (NSC 754633) against the full NCI cell lines panel derived from nine clinically isolated human cancer types described by three parameters: molar concentration of the compound causing 50% net cell growth inhibition (GI_50_), total growth inhibition (TGI), and 50% net cell death (LC_50_)

Subpanel	Cell line	GI_50_/μM			TGI/μM	LC_50_/μM
		Conc. per cell line	Subpanel MID^b^	SSR^d^	TGI-MID^e^	LC_50_-MID^f^
Leukemia			8.27	0.52	83.57	–^a^
	CCRF-CEM	3.08			–^a^	–^a^
	HL-60(TB)	12.9			–^a^	–^a^
	K-562	3.19			29.3	–^a^
	MOLT-4	3.69			72.1	–^a^
	RPMI-8226	23.4			–^a^	–^a^
	SR	3.33			–^a^	–^a^
Non-small cell lung cancer			4.52	0.94	31.10	71.33
	A549/ATCC	2.04			4.82	13.7
	EKVX	6.01			36.2	–^a^
	HOP-62	3.19			9.48	36.2
	HOP-92	3.14			–^a^	–^a^
	NCI-H226	5.07			25.0	–^a^
	NCI-H23	7.25			–^a^	–^a^
	NCI-H322M	7.82			90.8	–^a^
	NCI-H460	2.29			5.42	20.7
	NCI-H522	3.90			17.1	–^a^
Colon			6.70	0.64	36.74	64.73
	COLO 205	2.61			6.84	38.3
	HCC-2998	21.1			–^a^	–^a^
	HCT-116	4.01			16.1	71.1
	HCT-15	10.5			69.8	–^a^
	HT29	3.11			8.97	35.7
	KM12	3.69			51.6	–^a^
	SW-620	1.88			3.88	7.99
CNS cancer			3.24	1.32	12.68	36.39
	SF-268	2.07			5.32	23.4
	SF-295	3.40			14.6	51.4
	SF-539	3.40			3.95	27.1
	SNB-19	6.75			44.3	–^a^
	SNB-75	1.96			4.25	9.18
	U251	1.85			3.67	7.27
Melanoma			5.87	0.73	51.09	88.23
	LOX IMVI	3.09			–^a^	–^a^
	MALME-3 M	6.79			25.1	–^a^
	M14	4.51			–^a^	–^a^
	MDA-MB-435	2.90			10.8	75.0
	SK-MEL-2	2.64			8.73	49.2
	SK-MEL-28	5.50			21.4	69.9
	SK-MEL-5	2.91			10.3	–^a^
	UACC-257	13.6			83.5	–^a^
	UACC-62	10.9			–^a^	–^a^
Ovarian cancer			19.60	0.22	31.02	60.09
	IGROV1	10.2			39.0	–^a^
	OVCAR-3	2.29			4.37	8.34
	OVCAR-4	2.10			3.86	7.08
	OVCAR-5	16.4			46.6	–^a^
	OVCAR-8	2.55			7.74	36.6
	NCI/ADR-RES	–^a^			–^a^	–^a^
	SK-OV-3	3.65			15.6	68.6
Renal cancer			4.09	1.04	33.44	72.36
	786-0	2.64			6.47	–^a^
	A498	3.18			–^a^	–^a^
	ACHN	10.9			–^a^	–^a^
	CAKI-1	3.65			18.3	80.4
	RXF 393	2.39			5.14	14.6
	SN12C	3.57			15.5	75.3
	TK-10	2.68			5.77	34.6
	UO-31	3.68			16.3	74.0
Prostate cancer			5.44	0.78	56.15	84.05
	PC-3	7.54			–^a^	–^a^
	DU-145	3.33			12.3	68.1
Breast cancer			2.93	1.46	25.04	83.77
	MCF7	3.22			17.7	–^a^
	MDA-MB-231/ATCC	2.02			6.10	54.0
	HS 578T	2.26			7.04	–^a^
	BT-549	4.02			–^a^	–^a^
	T-47D	2.80			7.02	–^a^
	MDA-MB-468	3.30			12.4	48.6
MG-MID^c^		4.27			21.38	58.88

^a^Parameter not determined in five-dose assay, thus assumed 100 μM for the purpose of midpoint calculations

^b^Subpanel GI_50_ midpoint = average sensitivity of subpanel cell lines toward the test agent

^c^Mean-graph GI_50_, TGI, and LC_50_ midpoints = average sensitivity of all cell lines toward the test agent

^d^Subpanel selectivity ratio = subpanel MID:MG-MID

^e^Subpanel TGI midpoint

^f^Subpanel LC_50_ midpoint

COMPARE [38, 39] analysis at the NCI of compound **4c** showed moderate Pearson correlation coefficient (PCC = 0.446–0.549) with DNA interfering agents such as actinomycin D, echinomycin, bruceantin, chromomycin A3, or didemnin B (Table 4).

**Table 4:** COMPARE correlation coefficients (PCC) calculated using compound **4c** (NSC 754633) as seed, tested in US NCI-60 cell lines in vitro screen

Rank	NSC	Number of cell lines	PCC	Compd
1	3053	59	0.549	Actinomycin D
2	325014	58	0.547	Bactobolin
3	526417	56	0.520	Echinomycin
4	305884	58	0.517	Acodazole HCl
5	165563	56	0.511	Bruceantin
6	267469	58	0.493	Deoxydoxorubicin
7	58514	55	0.455	Chromomycin A3
8	325319	57	0.446	Didemnin B

For definitions and methods of calculation of the correlation coefficient from the COMPARE analysis, see Ref. [39]

## Conclusions

We designed a new and efficient method of obtaining substituted 2-mercaptobenzenesulfonamides from readily available 2,4-dichlorobenzenesulfonamides under optimized mild phase-transfer catalysis conditions. This approach offers easy and quick isolation of the products and preparative-scale synthesis. Novel 2-mercaptobenzenesulfonamides and their structurally diverse *N*-(hetero)aroyl derivatives were evaluated for in vitro antiproliferative activity. The discovered *N*-acylbenzenesulfonamide **4c** shows promising anticancer activity toward 50 human cancer cell lines and could be considered as a lead for further optimization.

## Experimental

Melting points were determined with a Boëtius apparatus. Infrared (IR) spectra were taken using a Thermo Mattson Satellite FTIR spectrophotometer, ^1^H and ^13^C nuclear magnetic resonance (NMR) were taken with a Varian Gemini 200 MHz or Varian Unity Plus 500 MHz spectrometer. Chemical shifts are reported in ppm ($\delta$). The results of elemental analyses for C, H, and N were in agreement with the calculated values within ±0.4% range. Column chromatography was carried out on silica gel Fluka Silica gel 60 (0.035–0.070 mm). The starting 2,4-dichloro-5-sulfamoylbenzhydrazide was obtained from commercially available 2,4-dichloro-5-sulfamoylbenzoic acid according to methods described previously [31].

### General procedure for the synthesis of **1a**, **1b**

A mixture of 2.84 g 2,4-dichloro-5-sulfamoylbenzhydrazide (10 mmol) and the appropriate orthoester (60 mmol) in 30 cm^3^ glacial AcOH was refluxed for 7–12 h. After cooling to room temperature, stirring was continued overnight. The precipitate was filtered off, washed with cold EtOH and petroleum ether, and purified by crystallization from EtOH.

#### *2,4*-*Dichloro*-*5*-*(1,3,4*-*oxadiazol*-*2*-*yl)benzenesulfonamide *(**1a**, C_8_H_5_Cl_2_N_3_O_3_S)

Starting from 8.89 g triethyl orthoformate. Yield: 2.42 g (82%); m.p.: 195–197 °C; *R*_f_ = 0.59 (benzene/EtOH = 4:1); IR (KBr): $\bar{\nu }$ = 3,323, 3,229, 3,165, 3,100, 1,359, 1,340, 1,168 cm^−1^; ^1^H NMR (200 MHz, DMSO-*d*_*6*_): $\delta$ = 7.97 (s, 2H, SO_2_NH_2_), 8.20 (s, 1H, H-3), 8.56 (s, 1H, H-6), 9.54 (s, 1H, Ar–H) ppm; ^13^C NMR (50 MHz, DMSO-*d*_*6*_): $\delta$ = 121.84, 131.12, 134.09, 134.56, 136.02, 140.83, 155.47, 160.89 ppm.

#### *2,4*-*Dichloro*-*5*-*(5*-*methyl*-*1,3,4*-*oxadiazol*-*2*-*yl)benzenesulfonamide* (**1b**, C_9_H_7_Cl_2_N_3_O_3_S)

Starting from 9.73 g triethyl orthoacetate. Yield: 2.13 g (69%); m.p.: 217–219 °C; *R*_f_ = 0.61 (benzene/EtOH = 4:1); IR (KBr): $\bar{\nu }$ = 3,305, 3,205, 3,094, 1,579, 1,542, 1,460, 1,354, 1,174 cm^−1^; ^1^H NMR (200 MHz, DMSO-*d*_*6*_): $\delta$ = 2.64 (s, 3H, CH_3_), 7.95 (s, 2H, SO_2_NH_2_), 8.18 (s, 1H, H-3), 8.51 (s, 1H, H-6) ppm; ^13^C NMR (50 MHz, DMSO-*d*_*6*_): $\delta$ = 10.93, 122.02, 130.75, 134.08, 134.21, 135.74, 140.77, 161.04, 165.08 ppm.

### General procedure for the synthesis of **2a**–**2d**

To a suspension of the appropriate 2,4-dichlorobenzenesulfonamide **1a**, **1b** (5 mmol) in 30 cm^3^ MeCN and 0.1 cm^3^ water, 1.52 g K_2_CO_3_ (11 mmol) and 0.016 g TBAB (0.05 mmol) were added. The obtained reaction mixture was vigorously stirred under an argon atmosphere, and slowly the appropriate mercaptan (5 mmol) was added dropwise. After 24 h of stirring at room temperature, the reaction mixture was concentrated under reduced pressure to dryness, and 15 cm^3^ EtOH was added. The precipitate was filtered off and suspended in 30 cm^3^ water, stirred for 30 min, and filtered off. The crude product was purified by crystallization from EtOH.

#### *2*-*Benzylthio*-*4*-*chloro*-*5*-*(1,3,4*-*oxadiazol*-*2*-*yl)benzenesulfonamide* (**2a**, C_15_H_12_ClN_3_O_3_S_2_)

Starting from 1.47 g **1a** and 0.62 g benzyl mercaptan. Yield: 1.55 g (81%); m.p.: 153–154 °C; *R*_f_ = 0.64 (benzene/EtOH = 4:1); IR (KBr): $\bar{\nu }$ = 3,435, 3,332, 3,142, 2,926, 1,590, 1,532, 1,495, 1,450, 1,350, 1,161 cm^−1^; ^1^H NMR (500 MHz, DMSO-*d*_*6*_): $\delta$ = 4.54 (s, 2H, SCH_2_), 7.29–7.32 (m, 1H, Ar–H), 7.36–7.39 (m, 2H, Ar–H), 7.52–7.54 (m, 2H, Ar–H), 7.73 (s, 2H, SO_2_NH_2_), 7.84 (s, 1H, H-3), 8.42 (s, 1H, H-6), 9.48 (s, 1H, Ar–H) ppm; ^13^C NMR (50 MHz, DMSO-*d*_*6*_): $\delta$ = 36.16, 118.23, 127.88, 128.89, 129.12, 129.61, 130.29, 135.19, 135.57, 139.55, 143.08, 155.18, 161.33 ppm.

#### *2*-*Benzylthio*-*4*-*chloro*-*5*-*(5*-*methyl*-*1,3,4*-*oxadiazol*-*2*-*yl)benzenesulfonamide* (**2b**, C_16_H_14_ClN_3_O_3_S_2_)

Starting from 1.54 g **1b** and 0.62 g benzyl mercaptan. Yield: 1.54 g (78%); m.p.: 208–210 °C; *R*_f_ = 0.67 (benzene/EtOH = 4:1); IR (KBr): $\bar{\nu }$ = 3,429, 3,246, 2,924, 2,854, 1,624, 1,591, 1,577, 1,558, 1,525, 1,495, 1,347, 1,165 cm^−1^; ^1^H NMR (500 MHz, DMSO-*d*_*6*_): $\delta$ = 2.58 (s, 3H, CH_3_), 4.50 (s, 2H, SCH_2_), 7.27–7.30 (m, 1H, Ar–H), 7.34–7.37 (m, 2H, Ar–H), 7.44–7.46 (m, 2H, Ar–H), 7.74 (s, 2H, SO_2_NH_2_), 7.78 (s, 1H, H-3), 8.35 (s, 1H, H-6) ppm; ^13^C NMR (50 MHz, DMSO-*d*_*6*_): $\delta$ = 10.87, 36.23, 118.89, 127.87, 128.70, 128.96, 129.14, 129.26, 129.33, 129.47, 129.60, 133.49, 135.70, 137.31, 145.04, 161.70, 164.32 ppm.

#### *4*-*Chloro*-*2*-*(4*-*chlorobenzylthio)*-*5*-*(1,3,4*-*oxadiazol*-*2*-*yl)benzenesulfonamide* (**2c**, C_15_H_11_Cl_2_N_3_O_3_S_2_)

Starting from 1.47 g **1a** and 0.79 g 4-chlorobenzyl mercaptan. Yield: 1.58 g (76%); m.p.: 185–187 °C; *R*_f_ = 0.63 (benzene/EtOH = 4:1); IR (KBr): $\bar{\nu }$ = 3,248, 3,156, 3,087, 2,918, 2,858, 1,589, 1,530, 1,490, 1,440, 1,350, 1,333, 1,162 cm^−1^; ^1^H NMR (500 MHz, DMSO-*d*_*6*_): $\delta$ = 4.55 (s, 2H, SCH_2_), 7.42–7.44 (m, 2H, Ar–H), 7.55–7.57 (m, 2H, Ar–H), 7.73 (s, 2H, SO_2_NH_2_), 7.84 (s, 1H, H-3), 8.42 (s, 1H, H-6), 9.48 (s, 1H, Ar–H) ppm; ^13^C NMR (50 MHz, DMSO-*d*_*6*_): $\delta$ = 35.29, 118.41, 128.84, 129.32, 130.29, 131.42, 132.49, 134.86, 135.20, 139.74, 142.55, 155.19, 161.29 ppm.

#### *4*-*Chloro*-*2*-*(4*-*chlorobenzylthio)*-*5*-*(5*-*methyl*-*1,3,4*-*oxadiazol*-*2*-*yl)benzenesulfonamide* (**2d**, C_16_H_13_Cl_2_N_3_O_3_S_2_)

Starting from 1.54 g **1b** and 0.79 g 4-chlorobenzyl mercaptan. Yield: 1.79 g (83%); m.p.: 250–252 °C; *R*_f_ = 0.68 (benzene/EtOH = 4:1); IR (KBr): $\bar{\nu }$ = 3,363, 3,239, 2,925, 2,853, 1,636, 1,587, 1,574, 1,559, 1,520, 1,493, 1,456, 1,349, 1,167 cm^−1^; ^1^H NMR (500 MHz, DMSO-*d*_*6*_): $\delta$ = 2.59 (s, 3H, CH_3_), 4.51 (s, 2H, SCH_2_), 7.41–7.43 (m, 2H, Ar–H), 7.47–7.49 (m, 2H, Ar–H), 7.76–7.77 (m, 3H, H-3 and SO_2_NH_2_), 8.35 (s, 1H, H-6) ppm; ^13^C NMR (50 MHz, DMSO-*d*_*6*_): $\delta$ = 10.87, 35.36, 119.03, 128.78, 128.94, 129.48, 131.27, 132.50, 133.51, 134.94, 137.44, 144.62, 161.67, 164.34 ppm.

### General procedure for the synthesis of 2-benzylthio-4-chloro-5-(1,3,4-oxadiazol-2-yl)-4-(*N*,*N*-dimethylamino)pyridinium *N*-acylbenzenesulfonamidates **3a**–**3c**

To the appropriate carboxylic acid (1.1 mmol) in 5 cm^3^ dry MeCN, 0.212 g 1-ethyl-3-(3-dimethylaminopropyl)carbodiimide hydrochloride (EDCI, 1.1 mmol) was added and stirred for 5 min. **2a** (0.382 g, 1 mmol) and 0.256 g DMAP (2.1 mmol) were added, and the reaction mixture was stirred at room temperature overnight. The precipitate was filtered off and washed with cold MeCN and MeOH. The crude salt was purified by crystallization from MeOH.

#### *4*-*(N,N*-*Dimethylamino)pyridinium 2*-*benzylthio*-*4*-*chloro*-*5*-*(1,3,4*-*oxadiazol*-*2*-*yl)*-*N*-*(pyrazine*-*2*-*carbonyl)benzenesulfonamidate* (**3a**, C_27_H_24_ClN_7_O_4_S_2_)

Starting from 0.137 g pyrazine-2-carboxylic acid. Yield: 0.338 g (55%); m.p.: 209–210 °C; *R*_f_ = 0.14 (benzene/EtOH = 4:1); IR (KBr): $\bar{\nu }$ = 3,198, 3,109, 3,056, 2,924, 1,646, 1,612, 1,589, 1,562, 1,498, 1,323, 1,142 cm^−1^; ^1^H NMR (200 MHz, DMSO-*d*_*6*_): $\delta$ = 3.17 (s, 6H, N(CH_3_)_2_), 4.35 (s, 2H, SCH_2_), 6.94–6.98 (m, 2H, Ar–H), 7.19–7.22 (m, 3H, Ar–H), 7.32–7.37 (m, 2H, Ar–H), 7.58 (s, 1H, H-3), 8.20–8.23 (m, 2H, Ar–H), 8.49 (s, 1H, H-6), 8.62–8.63 (m, 2H, Ar–H), 9.09 (s, 1H, Ar–H), 9.44 (s, 1H, Ar–H), 13.22 (br s, 1H, NH^+^) ppm; ^13^C NMR (50 MHz, DMSO-*d*_*6*_): $\delta$ = 35.76, 107.14, 117.12, 127.52, 127.67, 128.64, 129.25, 132.10, 133.50, 136.12, 139.51, 141.52, 143.23, 144.00, 145.22, 145.57, 150.80, 154.98, 157.13, 161.75, 167.83 ppm.

#### *4*-*(N,N*-*Dimethylamino)pyridinium 2*-*benzylthio*-*4*-*chloro*-*5*-*(1,3,4*-*oxadiazol*-*2*-*yl)*-*N*-*(pyridine*-*2*-*carbonyl)benzenesulfonamidate* (**3b**, C_28_H_25_ClN_6_O_4_S_2_)

Starting from 0.135 g pyridine-2-carboxylic acid. Yield: 0.219 g (36%); m.p.: 217–219 °C; *R*_f_ = 0.22 (benzene/EtOH = 4:1); IR (KBr): $\bar{\nu }$ = 3,195, 3,107, 2,924, 1,646, 1,607, 1,588, 1,562, 1,496, 1,324, 1,141 cm^−1^; ^1^H NMR (200 MHz, DMSO-*d*_*6*_): $\delta$ = 3.16 (s, 6H, N(CH_3_)_2_), 4.32 (s, 2H, SCH_2_), 6.91–6.95 (m, 2H, Ar–H), 7.18–7.21 (m, 3H, Ar–H), 7.31–7.32 (m, 2H, Ar–H), 7.41–7.45 (m, 1H, Ar–H), 7.57 (s, 1H, H-3), 7.78–7.86 (m, 1H, Ar–H), 7.94–7.98 (m, 1H, Ar–H), 8.23–8.26 (m, 2H, Ar–H), 8.50 (s, 1H, H-6), 8.55–8.57 (m, 2H, Ar–H), 9.45 (s, 1H, Ar–H), 13.20 (br s, 1H, NH^+^) ppm; ^13^C NMR (50 MHz, DMSO-*d*_*6*_): $\delta$ = 35.80, 107.05, 117.09, 123.74, 125.21, 127.49, 127.65, 128.63, 129.26, 132.30, 133.45, 136.04, 136.91, 139.89, 141.62, 143.21, 148.68, 154.99, 155.48, 157.00, 161.77, 169.37 ppm.

#### *4*-*(N,N*-*Dimethylamino)pyridinium 2*-*benzylthio*-*4*-*chloro*-*5*-*(1,3,4*-*oxadiazol*-*2*-*yl)*-*N*-*(thien*-*2*-*ylcarbonyl)benzenesulfonamidate* (**3c**, C_27_H_24_ClN_5_O_4_S_3_)

Starting from 0.141 g thiophene-2-carboxylic acid. Yield: 0.295 g (48%); m.p.: 201–202 °C; *R*_f_ = 0.16 (benzene/EtOH = 4:1); IR (KBr): $\bar{\nu }$ = 3,214, 3,090, 2,924, 1,649, 1,591, 1,565, 1,315, 1,138 cm^−1^; ^1^H NMR (200 MHz, DMSO-*d*_*6*_): $\delta$ = 3.17 (s, 6H, N(CH_3_)_2_), 4.33 (s, 2H, SCH_2_), 6.94–6.98 (m, 3H, Ar–H), 7.18–7.21 (m, 3H, Ar–H), 7.36–7.38 (m, 3H, Ar–H), 7.49–7.50 (m, 2H, H-3 and Ar–H), 8.18–8.22 (m, 2H, Ar–H), 8.44 (s, 1H, H-6), 9.43 (s, 1H, Ar–H), 13.18 (br s, 1H, NH^+^) ppm; ^13^C NMR (50 MHz, DMSO-*d*_*6*_): $\delta$ = 35.75, 107.20, 116.90, 127.31, 127.49, 128.66, 128.90, 129.04, 129.31, 129.56, 132.09, 133.14, 136.22, 139.51, 142.21, 143.27, 145.46, 154.93, 157.14, 161.81, 165.81 ppm.

### General procedure for the synthesis of *N*-acylbenzenesulfonamides **4a**–**4c**

To a suspension of the appropriate pyridinium salt **3a**–**3c** (0.5 mmol) in 5 cm^3^ EtOH, 2 cm^3^ 10% *p*-TSA solution in EtOH was added and stirred at room temperature for 1 h. The precipitate was filtered off and washed with EtOH and water.

#### *2*-*Benzylthio*-*4*-*chloro*-*5*-*(1,3,4*-*oxadiazol*-*2*-*yl)*-*N*-*(pyrazine*-*2*-*carbonyl)benzenesulfonamide* (**4a**, C_20_H_14_ClN_5_O_4_S_2_)

Yield: 0.242 g (99%); m.p.: 294–296 °C; *R*_f_ = 0.10 (benzene/EtOH = 4:1); IR (KBr): $\bar{\nu }$ = 3,485, 3,364, 3,298, 3,203, 2,871, 1,612, 1,585, 1,549, 1,492, 1,450, 1,362, 1,159 cm^−1^; ^1^H NMR (500 MHz, DMSO-*d*_*6*_): $\delta$ = 4.51 (s, 2H, SCH_2_), 7.08–7.17 (m, 3H, Ar–H), 7.29–7.31 (m, 2H, Ar–H), 7.89 (s, 1H, H-3), 8.58 (s, 1H, H-6), 8.81 (s, 1H, Ar–H), 8.94 (s, 1H, Ar–H), 9.08 (s, 1H, Ar–H), 9.47 (s, 1H, Ar–H) ppm; ^13^C NMR (50 MHz, DMSO-*d*_*6*_): $\delta$ = 35.98, 118.54, 127.81, 128.61, 129.27, 129.74, 133.91, 135.58, 136.99, 143.88, 144.09, 144.87, 148.89, 155.25, 161.03, 163.39, 163.44 ppm.

#### *2*-*Benzylthio*-*4*-*chloro*-*5*-*(1,3,4*-*oxadiazol*-*2*-*yl)*-*N*-*(pyridine*-*2*-*carbonyl)benzenesulfonamide* (**4b**, C_20_H_14_ClN_5_O_4_S_2_)

Yield: 0.241 g (99%); m.p.: 173–175 °C; *R*_f_ = 0.40 (benzene/EtOH = 4:1); IR (KBr): $\bar{\nu }$ = 3,138, 2,924, 2,854, 1,730, 1,647, 1,590, 1,530, 1,496, 1,450, 1,347, 1,174 cm^−1^; ^1^H NMR (200 MHz, DMSO-*d*_*6*_): $\delta$ = 4.47 (s, 2H, SCH_2_), 7.02–7.19 (m, 3H, Ar–H), 7.26–7.30 (m, 2H, Ar–H), 7.82 (s, 1H, H-3), 7.88–7.95 (m, 1H, Ar–H), 8.12–8.16 (m, 1H, Ar–H), 8.26–8.35 (m, 1H, Ar–H), 8.57 (s, 1H, H-6), 8.76–8.78 (m, 1H, Ar–H), 9.47 (s, 1H, Ar–H) ppm; ^13^C NMR (50 MHz, DMSO-*d*_*6*_): $\delta$ = 35.82, 118.20, 124.54, 127.67, 128.58, 128.79, 129.19, 129.30, 133.37, 135.73, 136.07, 136.66, 141.76, 143.52, 146.77, 147.41, 155.18, 161.20, 162.66 ppm.

#### *2*-*Benzylthio*-*4*-*chloro*-*5*-*(1,3,4*-*oxadiazol*-*2*-*yl)*-*N*-*(thien*-*2*-*ylcarbonyl)benzenesulfonamide* (**4c**, C_20_H_14_ClN_3_O_4_S_3_)

Yield: 0.244 g (99%); m.p.: 282–284 °C; *R*_f_ = 0.12 (benzene/EtOH = 4:1); IR (KBr): $\bar{\nu }$ = 3,382, 3,354, 3,253, 3,106, 1,614, 1,601, 1,579, 1,565, 1,549, 1,332, 1,318, 1,176 cm^−1^; ^1^H NMR (200 MHz, DMSO-*d*_*6*_): $\delta$ = 4.51 (s, 2H, SCH_2_), 7.30–7.76 (m, 12H, H-3 and Ar–H), 8.35 (s, 1H, H-6) ppm; ^13^C NMR (50 MHz, DMSO-*d*_*6*_): $\delta$ = 36.26, 118.90, 119.01, 126.03, 127.87, 128.46, 128.72, 128.96, 129.25, 129.50, 132.64, 135.75, 137.16, 137.65, 144.38, 155.74, 159.68 ppm.

#### *4*-*Chloro*-*2*-*(4*-*chlorobenzylthio)*-*5*-*(1,3,4*-*oxadiazol*-*2*-*yl)*-*N*-*(thien*-*2*-*ylcarbonyl)benzenesulfonamide* (**4d**, C_20_H_13_Cl_2_N_3_O_4_S_3_)

To a solution of 0.128 g thiophene-2-carboxylic acid (1 mmol) in 3 cm^3^ dry MeCN, 0.192 g EDCI (1 mmol) was added and stirred at room temperature for 5 min. **2c** (0.416 g, 1 mmol) and 0.184 g DMAP (1.5 mmol) were added and stirred at room temperature for 18 h. The reaction mixture was acidified with 2 cm^3^ 10% *p*-TSA/MeCN and concentrated under reduced pressure, and the residue was chromatographed with CH_2_Cl_2_/MeOH/AcOH (97:1:2) on silica gel column giving pure **4d**. Yield: 0.248 g (47%); *R*_f_ = 0.16 (benzene/EtOH = 4:1); m.p.: 205–207 °C; IR (KBr): $\bar{\nu }$ = 3,164, 3,094, 2,841, 1,678, 1,591, 1,526, 1,491, 1,450, 1,352, 1,262, 1,170 cm^−1^; ^1^H NMR (200 MHz, DMSO-*d*_*6*_): $\delta$ = 4.55 (s, 2H, SCH_2_), 7.08–7.12 (m, 2H, Ar–H), 7.23–7.27 (m, 1H, Ar–H), 7.32–7.37 (m, 2H, Ar–H), 7.44–7.46 (m, 1H, Ar–H), 7.90 (s, 1H, H-3), 7.97–8.00 (m, 1H, Ar–H), 8.53 (s, 1H, H-6), 9.48 (s, 1H, Ar–H) ppm; ^13^C NMR (50 MHz, DMSO-*d*_*6*_): $\delta$ = 35.02, 118.77, 128.57, 129.02, 129.97, 131.00, 131.35, 132.37, 133.00, 134.08, 134.90, 135.32, 135.43, 136.59, 136.95, 142.89, 155.23, 159.97, 160.99 ppm.

#### *2*-*Benzylthio*-*4*-*chloro*-*5*-*(5*-*methyl*-*1,3,4*-*oxadiazol*-*2*-*yl)*-*N*-*(thien*-*2*-*ylcarbonyl)benzenesulfonamide* (**4e**, C_21_H_16_ClN_3_O_4_S_3_)

To a solution of 0.128 g thiophene-2-carboxylic acid (1 mmol) in 3 cm^3^ dry MeCN, 0.192 g EDCI (1 mmol) was added and stirred for 5 min. **2b** (0.396 g, 1 mmol) and 0.184 g DMAP (1.5 mmol) were added and stirred at room temperature for 18 h. The obtained solution was concentrated under reduced pressure, and 2 cm^3^ 10% *p*-TSA/EtOH was added with vigorous stirring. The obtained suspension was left in the refrigerator overnight. The formed crystalline solid was filtered off and washed with cold EtOH. Yield: 0.213 g (42%); m.p.: 230–231 °C; *R*_f_ = 0.17 (benzene/EtOH = 4:1); IR (KBr): $\bar{\nu }$ = 3,098, 2,925, 2,854, 1,658, 1,591, 1,577, 1,525, 1,495, 1,453, 1,361, 1,176 cm^−1^; ^1^H NMR (500 MHz, DMSO-*d*_*6*_): $\delta$ = 2.62 (s, 3H, CH_3_), 4.50 (s, 2H, SCH_2_), 7.21–7.23 (m, 1H, Ar–H), 7.28–7.31 (m, 1H, Ar–H), 7.34–7.37 (m, 2H, Ar–H), 7.44–7.46 (m, 2H, Ar–H), 7.79 (s, 1H, H-3), 7.97–7.98 (m, 1H, Ar–H), 8.15–8.16 (m, 1H, Ar–H), 8.50 (s, 1H, H-6) ppm; ^13^C NMR (50 MHz, DMSO-*d*_*6*_): $\delta$ = 10.87, 36.39, 118.83, 127.98, 128.56, 129.02, 129.56, 132.56, 132.64, 133.06, 133.48, 135.35, 135.48, 136.27, 147.66, 160.11, 161.39, 164.43 ppm.

#### *4*-*Chloro*-*2*-*(4*-*chlorobenzylthio)*-*5*-*(5*-*methyl*-*1,3,4*-*oxadiazol*-*2*-*yl)*-*N*-*(thien*-*2*-*ylcarbonyl)benzenesulfonamide* (**4f**, C_21_H_15_Cl_2_N_3_O_4_S_3_)

To a solution of 0.128 g thiophene-2-carboxylic acid (1 mmol) in 5 cm^3^ dry tetrahydrofuran (THF), 0.206 g 1,3-dicyclohexylcarbodiimide (DCC, 1 mmol) was added and stirred for 5 min at room temperature. **2d** (0.430 g, 1 mmol) and 0.184 g DMAP (1.5 mmol) were added and stirred at room temperature for 48 h. By-products were filtered out and washed thoroughly with THF. The filtrate was acidified with 2 cm^3^ 10% *p*-TSA/EtOH and concentrated under reduced pressure, and the resulting oily residue was chromatographed with AcOEt/petroleum ether (1:1) on silica gel column giving pure **4f**. Yield: 0.135 g (25%); m.p.: 134–136 °C; *R*_f_ = 0.22 (benzene/EtOH = 4:1); IR (KBr): $\bar{\nu }$ = 3,422, 2,925, 2,855, 1,654, 1,575, 1,523, 1,490, 1,360, 1,261, 1,169 cm^−1^; ^1^H NMR (200 MHz, DMSO-*d*_*6*_): $\delta$ = 2.63 (s, 3H, CH_3_), 4.52 (s, 2H, SCH_2_), 7.20–7.24 (m, 1H, Ar–H), 7.39–7.51 (m, 4H, Ar–H), 7.77 (s, 1H, H-3), 7.97–7.99 (m, 2H, Ar–H), 8.15–8.17 (m, 1H, Ar–H), 8.51 (s, 1H, H-6) ppm; ^13^C NMR (50 MHz, DMSO-*d*_*6*_): $\delta$ = 10.58, 35.22, 118.62, 128.30, 128.48, 128.71, 131.06, 132.34, 132.76, 133.20, 134.27, 135.18, 135.97, 146.95, 159.82, 161.06, 164.15 ppm.

#### *2*-*Benzylthio*-*4*-*chloro*-*N*-*(5*-*chlorothien*-*2*-*ylcarbonyl)*-*5*-*(1,3,4*-*oxadiazol*-*2*-*yl)benzenesulfonamide* (**4g**, C_20_H_13_Cl_2_N_3_O_4_S_3_)

To a solution of 0.164 g 5-chlorothiophene-2-carboxylic acid (1 mmol) in 5 cm^3^ dry MeCN, 0.192 g EDCI (1 mmol) was added and stirred at room temperature for 5 min. **2a** (0.382 g, 1 mmol) and 0.184 g DMAP (1.5 mmol) were added and stirred at room temperature for 12 h. The obtained solution was acidified with 2 cm^3^ 10% *p*-TSA/MeCN and stirred under cooling (ice bath) for 2 h. The precipitated white solid was filtered off and purified by crystallization from MeCN. Yield: 0.268 g (51%); m.p.: 254–255 °C; *R*_f_ = 0.12 (benzene/EtOH = 4:1); IR (KBr): $\bar{\nu }$ = 3,160, 3,098, 2,924, 2,855, 2,717, 1,683, 1,592, 1,559, 1,531, 1,472, 1,351, 1,328, 1,168 cm^−1^; ^1^H NMR (500 MHz, DMSO-*d*_*6*_): $\delta$ = 4.51 (s, 2H, SCH_2_), 7.15–7.22 (m, 3H, Ar–H), 7.24–7.25 (s, 1H, Ar–H), 7.33–7.35 (m, 2H, Ar–H), 7.83 (s, 1H, H-3), 7.87 (s, 1H, Ar–H), 8.49 (s, 1H, H-6), 9.46 (s, 1H, Ar–H) ppm; ^13^C NMR (50 MHz, DMSO-*d*_*6*_): $\delta$ = 35.64, 118.22, 127.52, 128.39, 128.81, 129.01, 129.37, 132.56, 133.56, 134.98, 135.25, 135.75, 136.51, 136.62, 143.19, 154.91, 159.11, 160.73 ppm.

#### *2*-*Benzylthio*-*4*-*chloro*-*5*-*(1,3,4*-*oxadiazol*-*2*-*yl)*-*N*-*(pyridine*-*3*-*carbonyl)benzenesulfonamide* (**4h**, C_21_H_15_ClN_4_O_4_S_2_)

To a suspension of 0.135 g pyridine-3-carboxylic acid (1.1 mmol) in 5 cm^3^ dry MeCN, 0.212 g EDCI (1.1 mmol) was added and stirred for 5 min at room temperature. **2a** (0.382 g, 1 mmol) and 0.184 g DMAP (1.5 mmol) were added and stirred for 18 h at room temperature. The precipitate was filtered off, washed with MeCN, and then suspended in 1 cm^3^ EtOH, acidified with 1 cm^3^ 10% *p*-TSA/EtOH, and stirred for 2 h at room temperature. The precipitate was filtered off, washed with EtOH, and purified by extraction of contaminants with hot MeCN. Yield: 0.122 g (25%); m.p.: 282–284 °C; *R*_f_ = 0.30 (benzene/EtOH = 4:1); IR (KBr): $\bar{\nu }$ = 3,436, 3,096, 3,060, 2,926, 1,633, 1,589, 1,565, 1,520, 1,495, 1,355, 1,135 cm^−1^; ^1^H NMR (200 MHz, DMSO-*d*_*6*_): $\delta$ = 4.47 (s, 2H, SCH_2_), 7.20–7.33 (m, 5H, Ar–H), 7.73–7.78 (m, 2H, H-3 and Ar–H), 8.49–8.55 (m, 2H, H-6 and Ar–H), 8.85–8.87 (m, 1H, Ar–H), 8.09 (s, 1H, Ar–H), 8.47 (s, 1H, Ar–H) ppm; ^13^C NMR (50 MHz, DMSO-*d*_*6*_): $\delta$ = 35.88, 117.98, 125.11, 127.74, 127.96, 128.74, 129.29, 131.77, 133.19, 135.59, 135.76, 137.50, 139.95, 143.52, 146.76, 149.56, 155.14, 161.32, 164.82 ppm.

#### *2*-*Benzylthio*-*4*-*chloro*-*N*-*(4*-*chlorobenzoyl)*-*5*-*(1,3,4*-*oxadiazol*-*2*-*yl)benzenesulfonamide* (**4i**, C_22_H_15_Cl_2_N_3_O_4_S_2_)

To a solution of 0.172 g 4-chlorobenzoic acid (1.1 mmol) in 5 cm^3^ dry MeCN, 0.227 g DCC (1.1 mmol) was added and stirred at room temperature for 5 min. **2a** (0.382 g, 1 mmol) and 0.184 g DMAP (1.5 mmol) were added and stirred at room temperature for 72 h. By-products were filtered out and washed thoroughly with MeCN. The filtrate was concentrated under reduced pressure to dryness. MeOH (2 cm^3^) was added, and the obtained mixture was slowly acidified with 5 M hydrochloric acid. The formed precipitate was filtered off and washed with EtOH and water. The crude product was purified by crystallization from EtOH. Yield: 0.292 g (56%); m.p.: 275–277 °C; *R*_f_ = 0.25 (benzene/EtOH = 4:1); IR (KBr):$\bar{\nu }$ = 3,162, 3,080, 2,929, 2,854, 1,698, 1,592, 1,531, 1,492, 1,462, 1,348, 1,168 cm^−1^; ^1^H NMR (500 MHz, DMSO-*d*_*6*_): $\delta$ = 4.56 (s, 2H, SCH_2_), 7.14–7.21 (m, 3H, Ar–H), 7.32–7.36 (m, 2H, Ar–H), 7.57–7.62 (m, 2H, H-3 and Ar–H), 7.88–7.95 (m, 3H, Ar–H), 8.57 (s, 1H, H-6), 9.48 (s, 1H, Ar–H) ppm; ^13^C NMR (50 MHz, DMSO-*d*_*6*_): $\delta$ = 35.99, 118.53, 127.90, 128.80, 128.99, 129.36, 129.51, 130.41, 130.80, 134.00, 134.79, 135.39, 136.95, 138.61, 143.69, 155.24, 161.03, 164.52 ppm.

#### *2*-*Benzylthio*-*4*-*chloro*-*5*-*(1,3,4*-*oxadiazol*-*2*-*yl)*-*N*-*(3,4,5*-*trimethoxybenzoyl)benzenesulfonamide* (**4j**, C_25_H_22_ClN_3_O_7_S_2_)

To a suspension of 0.233 g 3,4,5-trimethoxybenzoic acid (1.1 mmol) in 5 cm^3^ dry MeCN, 0.227 g DCC (1.1 mmol) was added and stirred at room temperature for 5 min. **2a** (0.382 g, 1 mmol) and 0.184 g DMAP (1.5 mmol) were added and stirred at room temperature for 20 h. The precipitate was filtered off and suspended in 5 cm^3^ EtOH, acidified with 2 cm^3^ 10% *p*-TSA/EtOH, and stirred under cooling (ice bath) for 5 min. The crude product was filtered off and purified by crystallization from EtOH. Yield: 0.366 g (64%); m.p.: 245–247 °C; *R*_f_ = 0.29 (benzene/EtOH = 4:1); IR (KBr):$\bar{\nu }$ = 3,442, 3,158, 3,092, 2,962, 2,931, 2,841, 1,697, 1,595, 1,526, 1,511, 1,460, 1,331, 1,162 cm^−1^; ^1^H NMR (200 MHz, DMSO-*d*_*6*_): $\delta$ = 3.73 (s, 3H, OCH_3_), 3.78 (s, 6H, 2OCH_3_), 4.56 (s, 2H, SCH_2_), 7.16–7.36 (m, 7H, Ar–H), 7.90 (s, 1H, H-3), 8.59 (s, 1H, H-6), 9.49 (s, 1H, Ar–H) ppm; ^13^C NMR (50 MHz, DMSO-*d*_*6*_): $\delta$ = 36.01, 56.38, 60.45, 106.49, 118.49, 125.99, 127.87, 128.74, 129.39, 134.16, 134.79, 135.32, 136.94, 142.05, 143.73, 152.93, 155.25, 161.05, 164.81 ppm.

## NCI in vitro anticancer screen

As of early 2007 all compounds submitted to the NCI-60 cell screen are tested initially at a single high dose (10 μM) in the full NCI-60 cell panel representing human leukemia, melanoma and lung, colon, brain, breast, ovary, kidney, and prostate cancers. Briefly, the compounds were solubilized in DMSO and added at a single concentration, and the cell culture was incubated for 48 h at 37 °C, 5% CO_2_, 95% air, and 100% relative humidity. End points were determined by colorimetric sulforhodamine B (SRB) assay [40]. Results for each compound were reported as a mean-graph of the percent growth of the treated cells relative to the no-drug control, and relative to the time-zero number of cells. This allows detection of both growth inhibition (values between 0 and 100) and lethality (values less than 0) [41]. According to Developmental Therapeutics Program (DTP) anticancer screening paradigm, after obtaining the results for one-dose assay, careful analysis of DTP screening data was performed and compound **4c** (NSC 754633) which satisfied predetermined threshold inhibition criteria was selected for the NCI five-dose (0.01–100 μM) assay. The results were used to create dose–response curves (log_10_ of sample concentration versus % growth), and three response parameters (GI_50_, TGI, and LC_50_) were calculated for each cell line. GI_50_ measures the growth inhibitory power of the test agent, TGI signifies a cytostatic effect, and LC_50_ signifies a cytotoxic effect.
